# Accuracy of Geographically Targeted Internet Advertisements on Google Adwords for Recruitment in a Randomized Trial

**DOI:** 10.2196/jmir.1991

**Published:** 2012-06-20

**Authors:** Ray B Jones, Lesley Goldsmith, Christopher J Williams, Maged N Kamel Boulos

**Affiliations:** ^1^Faculty of Health, Education, and SocietyUniversity of PlymouthPlymouthUnited Kingdom; ^2^Institute of Health and WellbeingUniversity of GlasgowGlasgowUnited Kingdom

**Keywords:** Cluster randomized trial, contamination in RCTs, online advertising, depression, MoodGym, Living Life to the Full, Google Analytics, Google Adwords

## Abstract

**Background:**

Google AdWords are increasingly used to recruit people into research studies and clinical services. They offer the potential to recruit from targeted control areas in cluster randomized controlled trials (RCTs), but little is known about the feasibility of accurately targeting ads by location and comparing with control areas.

**Objective:**

To examine the accuracy and contamination of control areas by a location-targeted online intervention using Google AdWords in a pilot cluster RCT.

**Methods:**

Based on previous use of online cognitive behavioral therapy for depression and population size, we purposively selected 16 of the 121 British postcode areas and randomized them to three intervention and one (do-nothing) control arms. Two intervention arms included use of location-targeted AdWords, and we compared these with the do-nothing control arm. We did not raise the visibility of our research website to normal Web searches. Users who clicked on the ad were directed to our project website, which collected the computer Internet protocol (IP) address, date, and time. Visitors were asked for their postcode area and to complete the Patient Health Questionnaire (depression). They were then offered links to several online depression resources. Google Analytics largely uses IP methods to estimate location, but AdWords uses additional information. We compared locations assessed by (1) Analytics, and (2) as self-identified by users.

**Results:**

Ads were shown 300,523 times with 4207 click-throughs. There were few site visits except through AdWord click-throughs. Both methods of location assessment agreed there was little contamination of control areas. According to Analytics, 69.75% (2617/3752) of participants were in intervention areas, only 0% (8/3752) in control areas, but 30.04% (1127/3752) in other areas. However, according to user-stated postcodes, only 20.7% (463/2237) were in intervention areas, 1% (22/2236) in control areas, but 78.31% (1751/2236) in other areas. Both location assessments suggested most leakage from the intervention arms was to nearby postcode areas. Analytics data differed from postcodes reported by participants. Analysis of a subset of 200/2236 records over 10 days comparing IP-estimated location with stated postcode suggested that Google AdWords targeted correctly in just half the cases. Analytics agreed with our assessment that, overall, one-third were wrongly targeted by AdWords. There appeared little evidence that people who bothered to give their postcode did not answer truthfully.

**Conclusions:**

Although there is likely to be substantial leakage from the targeted areas, if intervention and control areas are a sufficient distance apart, it is feasible to conduct a cluster RCT using online ads to target British postcode areas without significant contamination.

**Trial Registration:**

Clinicaltrials.gov NCT01469689; http://clinicaltrials.gov/ct2/show/NCT01469689 (Archived by WebCite at http://www.webcitation.org/681iro5OU)

## Introduction

There is evidence that online health interventions can be effective [1], with online cognitive behavioral therapy offering effective treatment for anxiety and depression [2]. Packages such as MoodGYM [3] and Living Life To The Full [4] (LLTTF) are free and available globally. LLTTF is widely used in the United Kingdom and has been demonstrated to be effective, reducing depression for those recruited to use the online package [5], but we previously found a marked geographical variation in uptake. We analyzed distributions of self-reported postcodes of users of LLTTF in Britain over 1 year and found a 15-fold variation that was most likely explained by lack of awareness in some areas (see Multimedia Appendix 1). A Canadian study found lack of awareness to be the main barrier to effective use of online therapy for depression and other therapies [6].

Depression is a common condition that creates significant workloads for general practice and could be addressed at a population level. While primary care has a major role in health promotion, and some screening activities are run on a population basis from general practice, other public health initiatives have used mass media campaigns [7-9] and online advertising [10], but it is not clear how cost effective online promotion is. Targeted advertising is potentially important for clinical services wanting to contact people in their catchment areas, and in research also to allow geographically matched control arms to be identified.

While randomized controlled trials (RCTs) of online therapies can be carried out by recruitment and random allocation of individuals, the only rigorous way of comparing methods of raising awareness of online therapies is by geolocated cluster RCTs. Studies that look before and after an intervention at global level have no control group that can be adequately described and matched. So studies that simply recruit anyone on the Internet cannot safely match against a defined control area. As a consequence, any increase or decrease in uptake of a therapy could have happened for other reasons. For example, we know that mass media events (eg, a celebrity with a condition, or some other reason for a condition to be in the national news) may have an effect on uptake. Similarly, changes in health services, such as a rollout of measures to improve use of a therapy, or guidelines from a national body may have an impact in uptake nationally over time. Also, access to online therapies is affected by employment and deprivation rates [11]. Matching areas of intervention and control reduces the chances of bias. If we can limit online advertising to one area and compare it with another area where there is no advertising, then we can assume that any difference between the two geographical areas is due to the advertising. We can therefore estimate its cost effectiveness. Subsequently, we can decide whether it is worth using online advertising to raise awareness, or whether other methods would be more cost effective.

Google AdWords (Google Inc, Mountain View, CA, USA) provide the option of purchasing sponsored links that become visible when certain key words are targeted. They can be purchased to be targeted by location. AdWords have been used by others to recruit to studies, for example, to a depression screening site [12], for use of condoms [13], and to a quit-smoking campaign [14], but the cost effectiveness of their use was not assessed. To be able to carry out geolocated cluster RCTs, we need to know whether we can restrict the method of raising awareness to a particular area. If the intervention was via a group of general practitioners or through locally placed advertising, the contamination to other geographical areas is likely to be small. We have therefore run a pilot cluster RCT comparing AdWords targeting depression with local organization website ads and with no intervention, to increase the uptake of online cognitive behavioral therapy, in particular LLTTF [15].

Contamination between intervention and control arms is always of concern in RCTs and often one of the reasons for suggesting cluster rather than individual randomization. But cluster trials also have their limitations [16-21]. On the other hand, interventions, such as population-level advertising, can be randomized only at a cluster rather than an individual level. There seems to have been little examination of contamination between geographical clusters in this sort of trial. We needed to know whether it is possible to target online ads so that there is minimum leakage. Do services such as AdWords target locations as well as claimed? Can we be sure that normal online use of search engines does not corrupt the study? Will leakage be to adjacent areas or, given the increase in use of mobile access and other problems of geolocation, will it be random?

To our knowledge, this is the first study addressing the use of such approaches to target depression. We have assessed contamination between the two interventions that included online ads and control areas and discuss whether geographically targeted online interventions are possible for cluster RCTs.

## Methods

**Figure 1:**
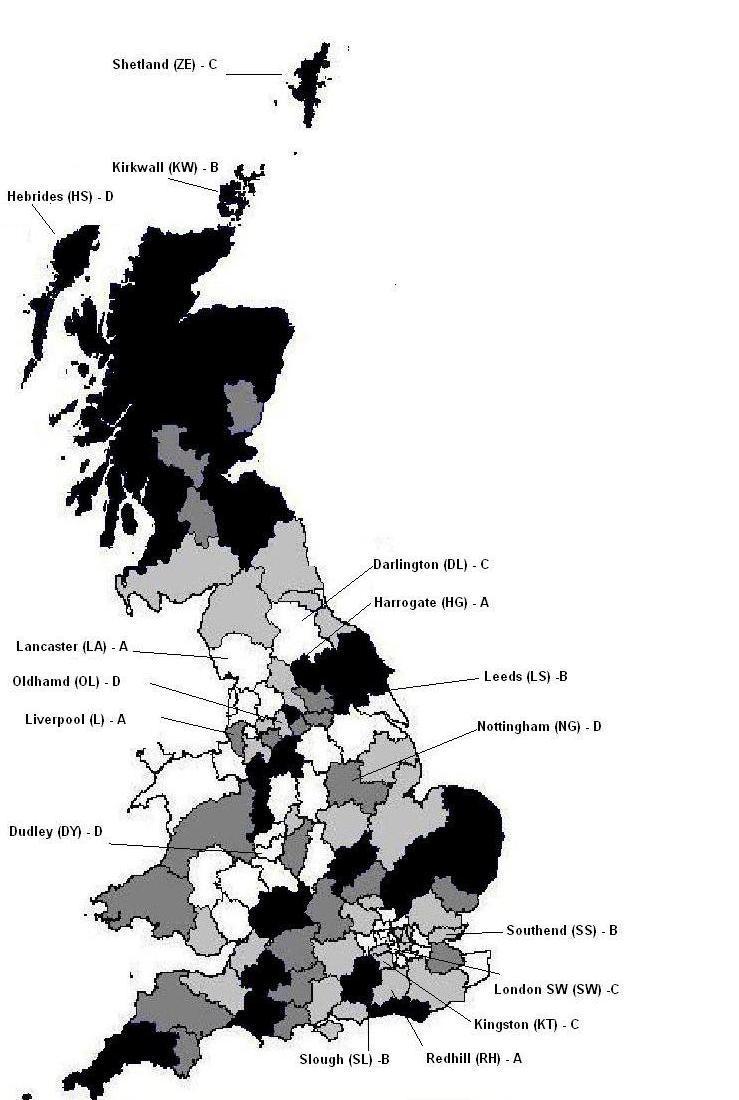
Registration rates by UK postcode area on Living Life To The Full between June 2008 and June 2009, showing four quartiles from darkest (top quartile) to white (lowest quartile) and 16 postcode areas (postcode letters; trial arm) sampled for study and randomized to four arms (A–D).

**Figure 2:**
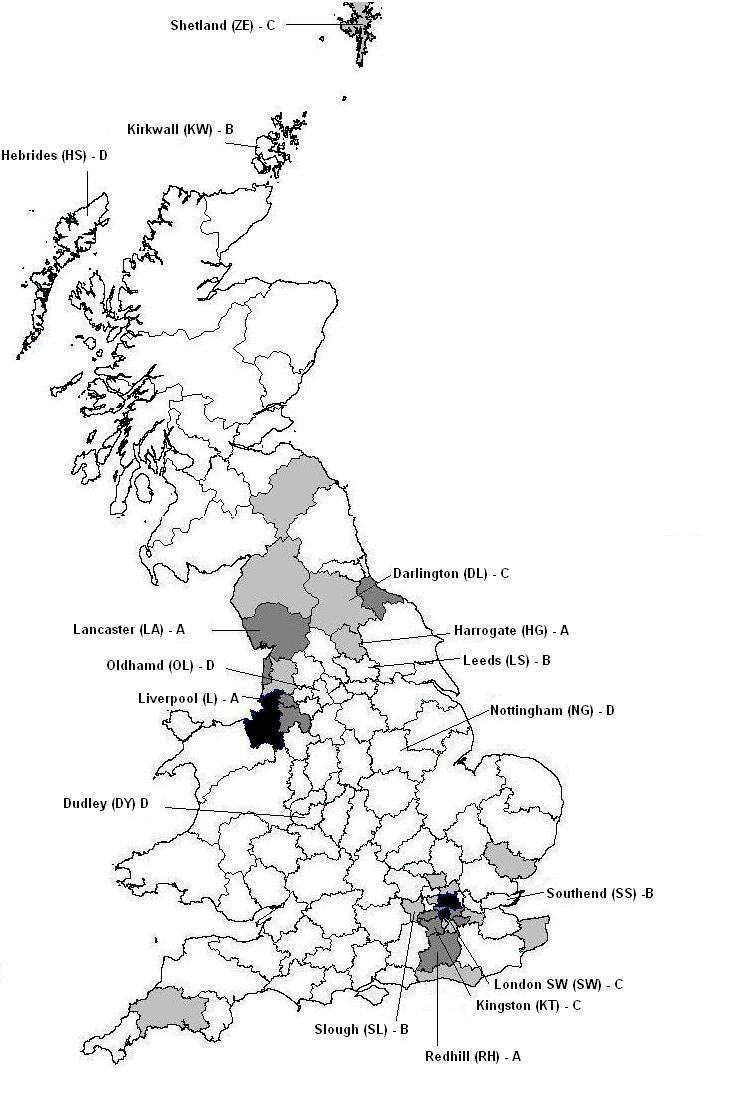
Postcode areas (as stated by participants) showing number of participants (out of 2236) expressed as a rate per million in four groups: darkest, 120+; dark gray, 80 to less than 120; light gray, 40 to less than 80; white, less than 40. Map shows the 16 postcode areas in the study, four in each of arms A, B, C, and D.

Previous registrants on LLTTF gave the first part of their postcode. We stratified postcodes according to their use of LLTTF and population (Figure 1 and Multimedia Appendix 1) and chose a purposive sample of 16 postcode areas that were similar in population and previous use of LLTTF but were, as far as possible, not adjacent. (Multimedia Appendix 2 shows adjacent postcode areas.) These were randomized to the four arms of the pilot cluster RCT (Table 1, Figure 2). In the eight areas in arms A and C we ran AdWords. In the eight arms in B and C we aimed to place ads on local organization websites such as local universities, general practices, and local authorities. Arm D was a control arm with no intervention. We had little success in placing ads on local websites, with only three sites (Leeds University, Leeds Carers, and Stronsay Limpet—arm B) agreeing in this period.

**Table 1:** Study areas, showing eight postcode areas (columns A and C) allocated to AdWords and eight postcode areas (columns B and D) allocated to another intervention and control (total population 7,000,564).

Study area characteristic	A (AdWords)	B (local websites)	C (AdWords and local websites)	D (control)
Total population	1,797,192	1,618,281	1,636,920	1,948,171
Postcode area	Liverpool (L)	Leeds (LS)	London SW (SW)	Nottingham (NG)
Estimated population^a^	843,450	737,343	783,340	1,080,230
Previous use of LLTTF^b ^(rate per 100,000 population)	57	68	56	59
Approximate distance (miles) to nearest control (center to center)	35 to Oldham	29 to Oldham	130 to Dudley	NA^c^
Postcode area	Redhill (RH)	Southend (SS)	Kingston (KT)	Oldham (OL)
Estimated population	494,414	493,206	490,104	443,800
Previous use of LLTTF (rate per 100,000 population)	39	46	52	43
Approximate distance (miles) to nearest control (center to center)	120 to Dudley	160 to Dudley	130 to Dudley	NA
Postcode area	Lancaster (LA)	Slough (SL)	Darlington (DL)	Dudley (DY)
Estimated population	325,972	337,631	341,488	397,639
Previous use of LLTTF (rate per 100,000 population)	31	23	38	32
Approximate distance (miles) to nearest control (center to center)	45 to Oldham	110 to Dudley	75 to Oldham	NA
Postcode area	Harrogate (HG)	Kirkwall (KW)	Shetland (ZE)	Hebrides (HS)
Estimated population	133,356	50,101	21,988	26,502
Previous use of LLTTF (rate per 100,000 population)	79	246	155	136
Approximate distance (miles) to nearest control (center to center)	50 to Oldham	135 to Hebrides	235 to Hebrides	NA

^a ^From Office for National Statistics. National Statistics Postcode Directory. November 2006. Version Notes; 2006. http://www.statistics.gov.uk/geography/downloads/NSPDVersionNotes.pdf.

^b ^Living Life To The Full.

^c ^Not applicable.

**Figure 3:**
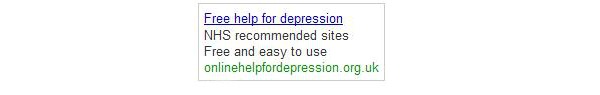
Location-targeted Google ad. NHS = UK National Health Service.

The Google ad (Figure 3) targeted eight postcode areas for two of the arms (A and C in Table 1) using AdWords-customized targeting. Targeting postcode areas (eg, KT) was not an option offered by Google. Options did, however, include targeting a radius of 1 mile or more around a postcode district (eg, KT2) or to hand draw a polygon to enclose the area of interest. Hand drawing a polygon to exactly cover postcode areas was quite difficult, so we used a mix of methods. Four postcode areas (HG, KT, RH, SW) were defined using circles of 1 mile radius for all postcode districts within the postcode area, and four (L, LA, DL, and ZE) were defined by hand-drawn polygons. Multimedia Appendix 3 shows the hand-drawn polygons, gives an assessment of how well the postcode district circles correspond to the postcode area boundary for London SW, and demonstrates the effect of this method in urban and rural areas.

We originally asked AdWords to display the ad for the keyword *depression*. AdWords suggested other similar keyword combinations and we accepted all suggestions (Multimedia Appendix 4). AdWords gives information on the number of impressions by day, keyword, and location. We ran one simple ad (Figure 3) for all locations with a maximum expenditure of £7.50 per day. AdWords decided when to present the ad. Users searching on terms such as *depression *and *depression help *would, depending on our budget and competing ads, be presented with our ad. Those who clicked on the ad were directed to our research website, which collected the computer Internet protocol (IP) address, date, and time. We specifically did not try to raise the visibility of our website to normal Google, Yahoo, or Bing searches.

Visitors were asked for their postcode area and to complete the Patient Health Questionnaire [22] assessing depression. Users were then offered four links: MoodGYM, LLTTF, NHS Choices information on depression [23], and Samaritans [24] (Multimedia Appendix 5). The order in which the links to MoodGYM and LLTTF were presented was randomized, and similarly the order of links to NHS Choices and Samaritans was randomized. Data collection methods and AdWords targeting were piloted and refined from April 17 to June 8, 2011. (Multimedia Appendix 6 gives details of some changes made.) We analyzed data from our website and Google Analytics (Google Inc) between June 9 and September 30, 2011 to evaluate the accuracy of targeting of the Google AdWords.

We used location data from Analytics (Table 2) and postcode areas as reported by users (Table 3) to estimate contamination between the intervention and control arms. Analytics is linked to AdWords to the extent that analysis can be restricted to people clicking through from an ad. We used users’ IP address as captured on our website to compare with their stated postcode as a comparison (Table 4).

We compared the location of the reported town from Analytics of people clicking on the ad within the 16 sample areas. Analytics has an idiosyncratic view of the geography of London, which seems to be reported as either London, Kensington, Lambeth, or Poplar, and various parts of greater London such as Wembley. It looks as if Google may have divided London into sections (West = Kensington, South = Lambeth, East = Poplar, North included as “London”) and then classified unknown places as “generic London.” However, despite our repeated attempts to obtain clarification, Google did not answer our requests to clarify this. Others have previously commented on this [25].

Analytics, like many other IP databases, returns a town name. We looked up each town given by Analytics on Google Maps, finding a postcode from the central area of that pin on the map. That postcode was deemed the face value area. We then put the postcode of that location into the Freemap Radius around a Point tool [26], plotting a 2 mile radius around that postcode. If the place name had a postcode that was within 2 miles of one of our sample areas, we allocated it to that area. Postcodes that were not in our sample were allocated to Other (Table 2).

We used the postcode area, as given by participants on our website questionnaire, to compare how many participants were in the intervention areas and how many in other areas, including our control areas (Table 3). We also calculated the rate of registration on our website for each postcode by dividing the number of registrations by the population of the relevant postcode area. Rates were divided into four groups and mapped, showing intervention and control areas (Figure 2).

For most analyses, we took the postcode area given by the registrant on our website as the reference standard. We had no definitive check on whether they entered their postcode area accurately, but we carried out a more detailed analysis of 200 consecutive users who completed our website questionnaire between September 1 and 10, 2011, comparing the location derived from their IP address and their stated postcode area. We compared the estimated location derived from the computer’s IP address with the stated postcode area using three IP location websites: (1) www.whatismyip.com, (2) www.whatismyipaddress.com, and (3) www.maxmind.com.

We examined agreement between the IP location methods and then between one of these (maxmind.com) and the stated postcode area. Combining these approaches, we estimated the impact of contamination on control areas.

The study was approved by the UK National Health Service (NHS) South West 2 Research Ethics Committee (Reference 11/H0203/8; February 2011).

**Table 2:** Google Analytics data showing total visitors (n = 3752) between June 9 and September 30 (by Cost per Click and other), classified using their town into an arm in the trial or as other area (ie, not in the trial).

Trial arm and intervention	Number of visitors (from Analytics)	
	n	%
A (AdWords)	554	15%
B (Website ads)	29	1%
C (AdWords and website ads)	2034	54.21%
D (Control)	8	0%
Other	1127	30.04%
Total	3752	

**Table 3:** Website data showing total visitors (n = 2236) between June 9 and September 30 (from all sources), classified using the postcode area they gave into an arm in the trial or as other area (ie, not in the trial).

Trial arm and intervention	Number of visitors (from stated postcode)	
	n	%
A (AdWords)	242	10.8%
B (Website ads)	47	2%
C (AdWords and website ads)	174	7.8%
D (Control)	22	1%
Other	1751	78.31%
Total	2236	

**Table 4:** Comparison of IP data with stated postcodes for a subsample of 200 visitors who completed our website questionnaire, including postcode area, between September 1 and 10, 2011.

		Stated postcode			
		Targeted	Adjacent	Elsewhere	Total
IP postcode	Targeted	16	7	3	26
	Adjacent	5	26	5	36
	London	10	24	50	84
	United Kingdom	3	4	11	18
	Elsewhere	3	10	23	36
	Total	37	71	92	200

## Results

**Figure 4:**
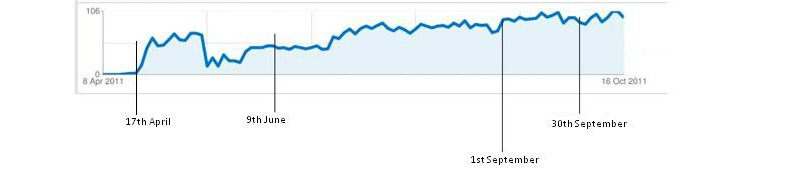
Google Analytics reporting of the number of clicks on the project website www.onlinehelpfordepression.org.uk every two days from start (April 2011) to mid-October 2011, showing period sampled for this study between June 9 and September 30.

Between June 9 and September 30, 2011, AdWords reported that the ad had been shown 300,523 times and the click-through rate was 1.4%, costing the project £848 at an average cost per click of £0.20. Analytics reported that we had 3929 unique visitors to the website making 4424 visits. Most (96.16%, 4254) visits were from search engines, of which 4207 (95.07%) were from AdWords. Only 37 visits were from normal searches using Google or Yahoo, 109 were direct traffic (the research team) not included in further analysis, 45 were from one referring site (Leeds University) and another 16 from other sites that were not part of the study. So the site had low visibility and very little access from the wider Web, apart from access by AdWords. Virtually all (99.21%, 4389) visits were from the United Kingdom, with the few overseas visits likely to be from national Web crawlers. A total of 12% (540) of visits were from mobile devices. The number of clicks on the site gradually increased over the study period (Figure 4).

Tables 2 and 3 show that in the period of study of just under 4 months we had 3752 UK visitors to the website, of whom 2236 (59.59%) completed the website questionnaire giving their postcode. Table 2, based on Analytics estimates of the locations of everyone visiting our website, suggests that there was very little contamination of the control arm in this cluster trial. In the worst-case scenario, only 22/3752 (1%) people who clicked on a Google ad were in the control area. On the other hand, the process was inefficient, as 30% of clicks were wasted on people who were not in the study areas. However, the postcode area stated by participants is our reference standard, and if we look at those who completed our website questionnaire and gave their postcode area (Table 3), we see that only 21% give a postcode in our study area and 1% were in the control area.

Figure 2 shows that most leakage from the intervention arms was to nearby or adjacent postcode areas and that control areas were far enough away not to have much contamination. More leakage seemed to be associated with Liverpool, Lancaster, and Darlington, where we had used polygons to define the areas (Multimedia Appendix 3), and in London SW.

Table 2 shows a major imbalance between the number of participants in arm C and arm A of the trial. Although the arm C intervention was intended to include local website referrals as well as AdWords, we were unable to place many such ads, so arm C was in effect simply AdWords. The target populations of arms A and C were similar, but arm C had nearly four times as many visitors according to Analytics. Table 3, on the other hand, shows that slightly more participants gave their postcode as belonging to arm A than to arm C, but with similar rates per 1000 population (0.13/1000 vs 0.11/1000, respectively).

We know (see above) that 95% of the visitors came to our website as a result of clicking on a Google ad. So, was the Google ad not well targeted or was the reporting by Analytics inaccurate, or did both contribute? Analytics suggests that 30% of people who clicked on an ad were not in the target areas. But, if we use the postcode areas provided by respondents, 80% were not in target areas (Table 3). However, we can see from Figure 2 that in many cases this was due to leakage to neighboring areas and that contamination of the control areas was only 1%.

To explore further why Analytics and the postcodes stated by participants gave a different picture, we compared the estimated locations from IP addresses. There was good agreement between two of the three location websites (187/200). The IP location given by www.whatismyip.com had little agreement either with the other two databases or with the stated postcode area. We have not used it further. As there was little difference between whatismyaddress and maxmind, we randomly chose maxmind to compare against the stated postcodes.

For this subsample of 200/2236 records over 10 days, 19% (37/200) of stated postcodes were in AdWords target areas, which is not dissimilar to that (21%) seen for the whole sample (Table 3). Half of the IP locations were vague (London or United Kingdom). Of the 34 (10 + 24) vague IP locations, 26/34 were for stated postcodes in or adjacent to London SW or Kingston and so could have been in agreement. For all but 1 of the 23 in which both IP location and stated location were off-target, the IP location and self-stated location were different. The 3 where the IP location was on-target and the 5 where the IP location was adjacent but the self-stated postcode was off-target may have been due to the person giving the wrong postcode (eg, their home address rather than current location). The best-case scenario for the accuracy of AdWords targeting is if we assume that the 108 people who gave their postcode as one of the target or adjacent postcodes were on-target or that there was a slight leakage. To this we might add the 8 people thought to be on-target or adjacent by the IP location but whose stated postcode was elsewhere. That is, in total, just over half may have had AdWords targeted correctly.

## Discussion

This study has shown that a cluster RCT of location-targeted online advertising using AdWords, randomizing at the postcode area level, in Britain is possible without too much contamination. In our pilot cluster RCT, we will ultimately compare the number of registrations on LLTTF in the intervention and control arms, and the associated costs. However, this intermediate, more detailed analysis of how location targeting works using data from AdWords, Analytics, and our website provides an insight into how to target online advertising by location for researchers considering an RCT using this method.

We had not identified in the literature any rigorous study of raising online awareness in mental health using a cluster RCT approach with geographical controls. This study has shown that, despite the limitations we encountered in using AdWords, cluster RCTs with geographical controls using British postcode areas are feasible. Contamination between intervention and control arms is not a major problem. In this study we used 12 postcode areas as interventions and 4 as controls, chosen from all 121 British postcode areas, and had less than 1% contamination. Provided the cluster design can have sufficient distance between intervention and control, leakage of location-based online ads should not significantly contaminate the control.

**Figure 5:**
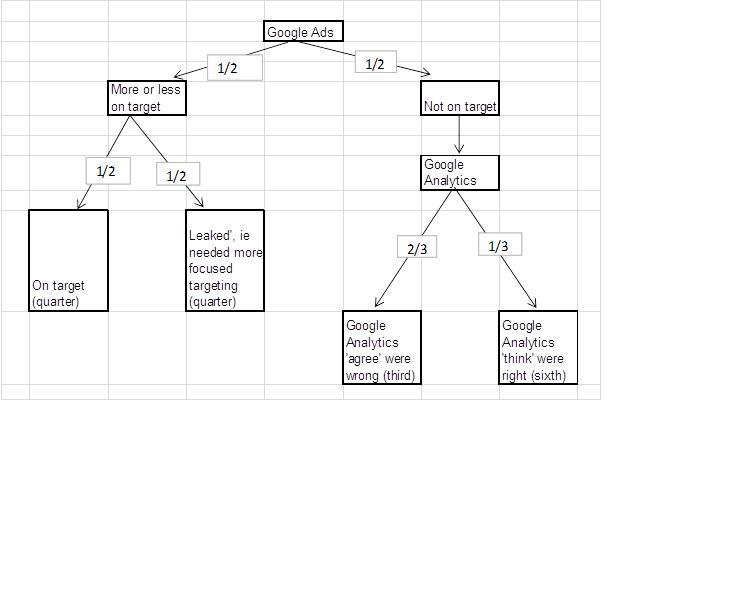
Approximate model explaining how the data we collected are consistent.

On the other hand, substantial leakage would increase the cost per successful contact. Because of the different denominators, sources of information, and ways of collecting and classifying locations, interpreting Tables 2–4 is quite difficult. At first sight, the numbers may appear contradictory. Figure 5 shows a model of our interpretation of these data. From Table 4, we concluded that about half of the online ads were roughly on-target. We approximated figures from Table 3 to conclude that a quarter were exactly on-target and a quarter had leaked. Overall reporting by Analytics suggested that two-thirds of ads were correctly geographically targeted (Table 2); this implies that Analytics “thinks” that two-thirds to one-half of those off-target are actually right. Thought of in terms of sensitivity and specificity, our campaign was specific, in that no more than 1% (Tables 2) of visitors to our website were from control areas, but was not very sensitive, in that only a quarter (Figure 5) were in target areas.

AdWords currently uses a composite method of IP or user’s address, Web history, and other clues, while Analytics considers only the visitor’s IP address and a lookup database. Bearing this in mind, Table 2 shows that although we asked AdWords to target certain areas, by Google’s own Analytics reporting, it failed in 30% of cases. As Figure 5 shows, some of this failure can be explained by leakage—that is, participants who could have been in the targeted areas if we had used the available methods better—so we cannot blame AdWords for getting it wrong. We estimate that AdWords got the geographical targeting right for about half the people who saw the ads—that is, about half of our participants were actually in the target postcodes when they clicked on the ad. Of the half whom AdWords did not get on-target, Google (via Analytics) “admitted it” for two-thirds—that is, Analytics recorded them as being in areas that were not targeted by AdWords—whereas Analytics and AdWords were consistent (but wrong) for one-third of those not on-target (ie, one-sixth overall).

Despite the wasted ads, the cost per participant within our intervention areas (approximately £1/person) is still low relative to other methods of raising awareness and is likely to be much more cost effective. For example, in another strand of research in secondary care [27], and more recently in primary care [28], we have been recruiting patients to offer them help in using the Internet. Leafleting methods, particularly in general practice or community settings, cost tens or even hundreds of pounds per person recruited. Of course, if we were not carrying out a cluster trial, AdWords would appear even more cost effective, as raising awareness of online cognitive behavioral therapy is useful wherever people live. The cost of AdWords depends on current competition for the particular keywords being used, so the costs for studies would be different.

The English Department of Health was heavily criticized for spending £2.5 million on AdWords between February 2009 and January 2010 [29]. The Department of Health declined to give further details of how they had spent this money or its cost effectiveness. By judicious design of the website, NHS Choices and other NHS websites should be a high-profile search result. If a Google search returns a website among its first page of normal search results, then advertising using AdWords may be a waste of money. When there is substantial competition for a user’s attention, advertising may be worthwhile. The results of a search and the additional impact of advertising should be explored in more detail before committing to a long-term advertising budget. For example, we examined the probability of finding online cognitive behavioral therapy sites by searching for the term *depression *and have estimated the increased probability as a result of our AdWords campaign (paper submitted). It is possible that the NHS AdWords campaign may have been implemented without prior piloting or evaluation. It may have been cost effective but we do not know.

### What Have We Learned?

Have we learned helpful points about using AdWords (in its current format) that may be of use to other researchers? Arm C, which included London SW, had (according to Analytics) nearly four times the number of registrants on our website compared with arm A. The population of each was similar, so we must hypothesize that (1) AdWords showed the ad disproportionately (for a given number of people searching on *depression*) more often in London, resulting in more hits, or (2) there were more people in London searching on *depression*, resulting in AdWords displaying it more often, or (3) there was more competition for ads in areas other than London, or (4) more people in London than in other areas clicked through on the ad.

On the other hand, when we examined our website data, we did not see this same imbalance, suggesting two further explanations: (5) that AdWords worked quite well in targeting on the requested areas but Analytics reported very badly, or (6) that many more people from London than from other regions logged on to the site but did not complete the questionnaire. AdWords supplies the number of impressions only by keyword and not by area for a given campaign, and Analytics does not give information on the number of impressions. We do not know how AdWords decides to display ads if it is given a choice of regions for a given budget.

**Figure 6:**
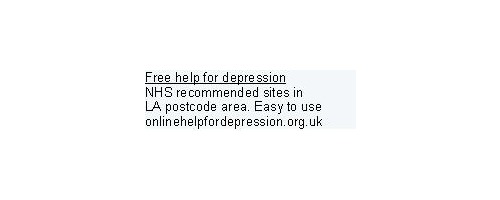
Alternative Google ad for the LA (Lancaster, UK) postcode area.

AdWords provides click-through rates by campaign or by keyword but not by location within a campaign. As we had our AdWords campaign set up initially—as one campaign that included the various locations—we could not analyze click-through rate by location. In retrospect, a better strategy to running one AdWords campaign with multiple postcode areas would be to run separate campaigns for each of the target postcode areas with their own separate budgets allocated proportionately to their populations. This would give more information about why ads were performing better in parts of an intervention arm and would allow for greater control. Furthermore, the postcode area could be included in the ad wording to help in location targeting (Figure 6). After completing data collection for this study, we ran regional ads of this sort for 2 months. Data presented in Multimedia Appendix 7 show that AdWords presented the ad more than twice as often in London SW as in Liverpool per head of population than in other areas. There was no major difference in click-through rates. Running separate campaigns in each area appears to be a better strategy in being able to control a study, but questions remain as to why there should be such a large variation in presentation of ads.

Another alternative is to consider excluding London postcodes from the study. However, this would mean that the capital, with its different population and environment compared with many parts of Britain, would not be represented. London distorts the targeting, perhaps because of the likelihood that Internet service providers are based in London or because of the idiosyncratic way that IP location databases and Google interpret the geography of London [25]. Whatever strategy is adopted, the imbalance in the arms points to the need to pilot the study design.

When we started this study, AdWords offered targeting through drawing a radius around a point and a hand-drawn polygon. We used both methods but probably had more leakage from using polygons. Google has (as of October 29, 2011) abandoned the use of hand-drawn polygons [30]. Given our experience, in future (1) we would use only the radius method with the smallest radius possible (1 km) rather than 1 mile in urban areas, (2) in urban areas, we might consider targeting the center of each sample area, leaving a no-man’s-land of untargeted areas near the postcode area boundary, and (3) in rural areas, on the other hand, we might use larger-radius circles, as the surface area of postcode districts varies greatly between rural and urban areas (see Multimedia Appendix 3).

### Recommendations for Future Trials

In summary, we would recommend running separate campaigns for different areas with budgets proportional to the target populations, so that the number of presentations of ads and click-through rates can be more easily monitored. We would use radius around a point rather than hand-drawn polygons, and would use the smallest radius (1 km) possible in urban areas. We might avoid including London in a trial and we would put more emphasis on distance between areas included in the study, in particular, to control areas, and less emphasis on trying to match on other characteristics.

### Considerations for Improvements in AdWords and Analytics to Support Research

Supporting health research is not Google’s main priority, but if we were able to see improvements in Google products, what would be useful for studies of this type? First, we would like AdWords and Analytics to coordinate better. For example, if we request AdWords to target an area X, we would expect Analytics reports to show that area X was targeted. Table 2 shows that this is currently not the case. Others have commented on the different systems used by AdWords and Google Places [31]. Second, we would like to be able to understand the geography used, particularly of London. Third, we would like more integration between Google Insights and Google AdWords, so that we could understand better whether getting more presentations of an ad in one area was due to the volume of users searching on relevant terms or some function of competing bids.

### Limitations

There is of course no guarantee that the ability to target online advertising will always be available in this form. While the increasing use of mobile access to the Internet is encouraging the use of location-specific ads [32], concerns about privacy (eg, [33,34]) and the changing legal environment have resulted in Google allowing people to opt out of its location service [35]. In this study, 12% of visits were from mobiles; it is possible that as this proportion increases, locating users may become more difficult, resulting in more leakage.

AdWords is of course not the only way of local advertising online. This is a complex domain with large financial gains or losses to be made between the big corporations. Ongoing comparisons of the various geotargeted advertising solutions from Microsoft, Facebook, LinkedIn, etc would be worthwhile. For example, Facebook allows geotargeting of ads by radius and other useful criteria such as age, demographics, and interests [36]. Microsoft is now offering radius targeting for Bing and Yahoo with a radius of 5 to 100 miles [37]. Ongoing comparisons are particularly important if we are to ensure that the appropriate population is targeted for a study.

Google has 60%–80% of the search market globally [38]. Facebook is used by 43% of the UK population [39], although it may be underrepresented among, for example, older people or people with few social contacts (who may be the population that most needs to be targeted). Google is starting to compete with Facebook by promoting Google+ [40], where users can volunteer as much information as they wish about themselves and their current and past locations, and so Google may revise and refine its geotargeting strategy in due course. Each company will continue to develop its methods, sometimes leading to improved, sometimes to reduced, options for researchers.

Cluster trials are stronger—that is, less subject to chance bias—if they include more clusters [21]. Our study, although selection of our clusters took into account population size and previous use of LLTTF, was therefore relatively weak in that each arm included only four postcode areas (clusters). There are two ways we could have included more clusters. First, we could have included more postcode areas. Britain has 121 postcode areas, of which 16 were in our study. Given the shape of Britain, the shape of the postcode areas, the fact that we purposively sampled by quartiles of previous use of LLTTF and population size, and that we had four arms, we already had some difficulty in selecting areas that were not adjacent. However, a simpler trial with two arms and more areas sampled should be possible and would give a stronger design. In a larger country such as the United States it may be easier to choose control arms with no contamination.

The alternative way of increasing the number of clusters is to reduce the target size to a postcode district—that is, to target, for example, KT1, SW4—with controls at the district level. Britain has just over 3000 postcode districts such as this. In our use of AdWords, we defined postcode areas by the sum of postcode districts. Leakage between postcode districts within an area (eg, between KT1 and KT2) was not a problem for our design, in that leakage was into another district in the same geographical area. If we had used single postcode districts scattered over Britain, there would have been more leakage into non-study areas and it would have been difficult to find suitably distant control areas. There is clearly a trade-off between reducing the bias from having a small number of clusters and having leakage on a greater number of clusters. Analysis of registration on LLTTF by postcode district will allow assessment of intracluster correlation. We think that postcode area is the best size of region for this type of study in the United Kingdom, but having only two arms with more areas in each would be preferable.

We cannot be sure how the findings of this study would translate to other countries, particularly in relation to the complications of including London described above. It seems quite likely that densely populated cities that host many Internet service providers may cause some distortion of the geography used by online advertising.

Other limitations to this study relate to the lack of information from Google or inconsistencies in methods. For example, in trying to assess leakage, we used Google Maps to pin a location for a town and to subsequently draw a radius and assess whether the area of someone using our website might have been targeted by AdWords. But, as we have seen, Google is not consistent between Ads and Analytics, or between Ads and Places, so is quite likely also not to be consistent between Maps and Analytics. However, in the absence of an integrated system, this method did give us an idea of whether we could attribute a case to leakage or to random error.

### Conclusions

At least for now, it seems feasible to carry out a cluster RCT of location-targeted online ads in Britain at the postcode area level. Internet recruitment provides unique challenges in understanding the characteristics of participants and which denominators and populations to use [41]. We have highlighted several issues that may help other researchers to use AdWords and other online advertising, but we conclude that it is possible to geographically target Internet ads in a cluster RCT without too much contamination.

## Multimedia Appendix 1

How the postcode area samples were chosen.

## Multimedia Appendix 2

Adjacent areas to the sampled postcode areas.

## Multimedia Appendix 3

Further information on boundaries.

## Multimedia Appendix 4

Keywords.

## Multimedia Appendix 5

Screen shots from research website.

## Multimedia Appendix 6

Change made as a result of the pilot period.

## Multimedia Appendix 7

Google Adwords budget, clicks, and impressions in two phases of the study.

## Abbreviations

IP: Internet protocol
LLTTF: Living Life To The Full
NHS: UK National Health Service
RCT: randomized controlled trial
