# Supplementary material for: Accuracy of Geographically Targeted Internet Advertisements on Google Adwords for Recruitment in a Randomized Trial
Source: J Med Internet Res. 2012 Jun 20;14(3):e84. doi: 10.2196/jmir.1991 (PMC3414907; doi:10.2196/jmir.1991)
Supplement: Supplementary file 1 [file jmir_v14i3e84_app1.pdf]

APPENDIX 1: How the postcode area samples were chosen

This appendix gives further details of why we are assessing the role of online advertising in a cluster randomised trial. It gives further details of how the postcode area samples were chosen.

BACKGROUND

Anecdotally it seemed that LLTTF was used in some areas, particularly Scotland, but not others. We audited routinely collected data from LLTTF to see if this was the case.

METHODS

Numerators: People registering to use LLTTF from 16th June 2008 to 14th June 2009 were asked to complete a Hospital Depression and Anxiety Score (HADS) [1] and to give the first part of their postcode. Those with HADS scores of 8 or more on either depression or anxiety were included in the analysis of postcodes.
Denominators: A CD of populations by postcode from the 2001 census was obtained from Office for National Statistics (ONS) . This contained head counts for each of the 1,296,799 postcode output areas for England and Wales. These were summed into populations for 106 postcode areas (i.e. first two characters of the postcode). Postcode areas (121) for England, Wales and Scotland were taken from ONS [2] to check and collate population figures. Population figures for Scotland by postcode were obtained from the MRC unit in Glasgow (personal communication, Harper Gilmour, University of Glasgow); the total figure corresponded with that published by ONS [3].
Registration ratios: We calculated a standardised ‘registration ratio’ for each postcode area by dividing the observed count of registrations by the total population count in each area. We present the distribution and a map of registration rates by postcode area.
How they heard of LLTTF: We used routine statistics collected by LLTTF in 2007 on registration, asking new registrants how they learned about LLTTF,

RESULTS

How did users of LLTTF learn about it? 24% found out about LLTTF from website links, 21% from their GP, 15% from friends, 11% from self help groups, 8% from the media, and only 6% from search engines.
Geographic variation in prevalence: The one year registration rate for Scotland varied between 39/100,000 (Dumfries) and 246/100,000 (Kirkwall). Overall the rate in Scotland was 6084/5062010, i.e. 120 per 100,000, while the one year registration rate for England and Wales was 29200/52,025,448, i.e. 56 per 100,000. The Scottish rate was over twice that of England and Wales (F=19.6, 118 df, $p<0.001$).
For the 104 English postcodes, rates per 100,000 population varied from 16-195, mean 55 (SD 26) (Figure 1). Only one English area (Ipswich, postcode area IP) had a rate greater than the Scottish mean. The rate in Ipswich was 195/100,000; excluding this outlier the mean rate per 100,000 in England and Wales was 54 (SD 22). Table 1 shows the number of registrants and the rates per 100,000 in rank order for English and Scottish postcode areas.

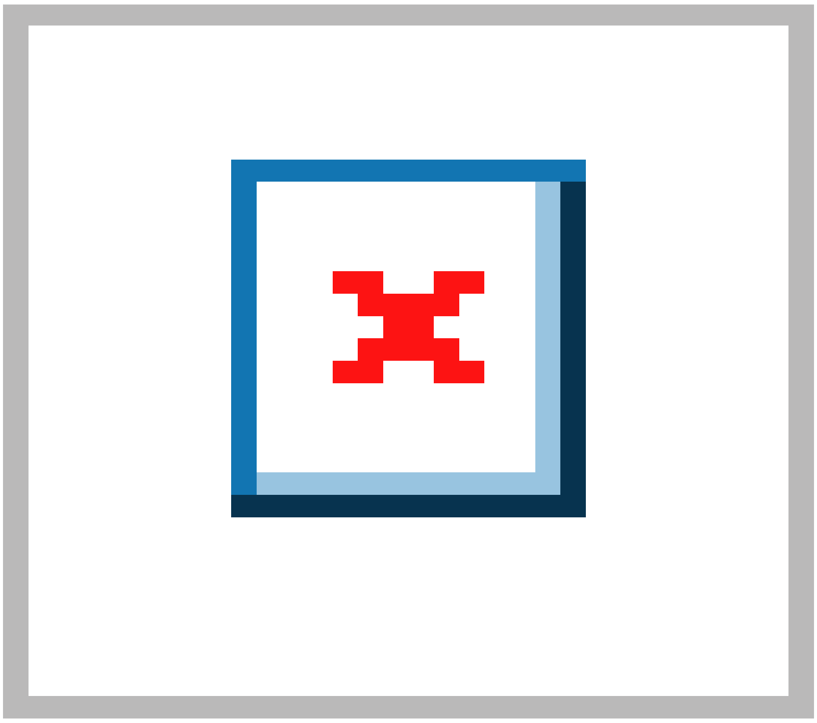

Figure 1. Distribution of registration rates by postcode area, showing English/Welsh and Scottish postcode areas.

Rate	Scotland	England and Wales
246	Kirkwall	
190-195	Edinburgh	Ipswich
136-163	Inverness, Shetland, Paisley, Hebrides	
124-128	Glasgow, Perth Aberdeen	Norwich
91-111		Northampton, Guildford, Dorchester, Cambridge, Halifax, Brighton, Stockport
80-89	Kilmarnock, Kirkcaldy Galashiels	Truro, Gloucester, Bath, Plymouth, Telford, Crewe
70-79	Dundee	Harrogate, Stevenage, York, St Albans, Watford, Swansea, Torquay, Dartford, Bristol, Oxford
60-69	Falkirk	Salisbury, Milton Keynes, Leeds, London EC, Exeter, Bournemouth, Manchester, London W, Birmingham, Shrewsbury, Wakefield
50-59	Motherwell	Nottingham, Colchester, Medway, Liverpool, Blackpool, London SW, London SE, Huddersfield, Swindon, Cleveland, Portsmouth, Durham, Walsall, Kingston upon Thames, Southampton, Reading, London N
40-49		Lincoln, Peterborough, Cardiff, Warrington, Newcastle upon Tyne, Luton, Southend-on-Sea, Bradford, London WC, Carlisle, Tonbridge,

		Romford, London E, Leicester, Oldham, Chelmsford, Hemel Hempstead, Taunton, Twickenham, Bolton
30-39	Dumfries	Bromley, Redhill, Darlington, Derby, Sheffield, Sunderland, London NW, Stoke-on-Trent, Canterbury, Chester, Worcester, Coventry, Sutton, Dudley, Lancaster, Wolverhampton, Harrow, Blackburn, Preston
20-29		Llandrindod Wells, Croydon, Llandudno, Southall, Doncaster, Hull, Newport, Ilford, Enfield, Slough
16-19		Hereford, Wigan

Table 1. English/Welsh and Scottish registration rates per 100,000 showing postcode areas in rank order.

DISCUSSION

Why did LLTTF show such large geographical variation?

It was unlikely to be (solely) because of variation in Internet access. By August 2009, 70% of homes had Internet access [4]. Although there was still variation by region (for example, in 2007 60% of households in London compared to 40% in Northern Ireland had a broadband connection), the main variation in Internet use was, and still is, by age [4]. It was also unlikely to be (solely) because of geographical variation in the prevalence of depression. Variation in the prevalence of depression at postcode area is likely to be small [5].

Although there may also be geographical preferences in the use of different CCBT packages, (e.g. some areas may have predominantly used MoodGym), a Canadian doctoral study found lack of awareness to be the main barrier to effective use of online therapy for depression and other therapies [6] and it seemed likely that this was the case in the UK as well. The most likely explanation for variation in LLTTF registration was lack of awareness by people with depression or resistance to recommending CCBT by health professionals.

Whatever the cause, it seemed worthwhile to raise awareness of CCBT. Although there are a variety of ways of raising awareness of CCBT including via GPs and via the mass media, we thought that online methods were likely to be the most cost effective and for this reason we are carrying out a pilot cluster randomised trial of online methods to raise awareness of CCBT, in particular Moodgym and LLTTF.

The current paper is a preliminary step towards assessing the feasibility of carrying out a cluster trial of online advertising. The main paper describes the sampling used for the 16 study areas.

References

1. Zigmond AS, Snaith RP. The Hospital Anxiety and Depression Scale. Acta Psychiatr Scand 1983; 67(6): 361-370.

2. Office for National Statistics, National Statistics Postcode Directory. November 2006. Version Notes. 2006.

3. Office for National Statistics. *Population Pyramids. Census 2001*. URL:http://www.statistics.gov.uk/census2001/pyramids/pages/179.asp. Accessed: 2011-11-04. (Archived by WebCite® at http://www.webcitation.org/62wQVwMbs)

4. Office for National Statistics. *Internet Access - Households and Individuals, 2011*. URL:http://www.ons.gov.uk/ons/publications/re-reference-tables.html?edition=tcm%3A77-226727.. Accessed: 2011-11-04. (Archived by WebCite® at http://www.webcitation.org/62wQjFRnd)

5. Weich S, Holt G, Twigg L, Jones K, Lewis G. Geographic Variation in the Prevalence of Common Mental Disorders in Britain: A Multilevel Investigation. Am. J. Epidemiol 2003; 157(8): 730-737. PMID:12697577

6. Kakuma R. Utilization of health services for depression and anxiety in Ontario: An eleven-year comparison of determinants. 2007. McGill University: Montreal. p. 278. URL:http://digitool.library.mcgill.ca/R/?func=dbin-jump-full&object_id=19260&local_base=GEN01-MCG02. Accessed: 2011-11-04. (Archived by WebCite® at http://www.webcitation.org/62wRrk4dS)
