# Supplementary material for: Accuracy of Geographically Targeted Internet Advertisements on Google Adwords for Recruitment in a Randomized Trial
Source: J Med Internet Res. 2012 Jun 20;14(3):e84. doi: 10.2196/jmir.1991 (PMC3414907; doi:10.2196/jmir.1991)
Supplement: Supplementary file 2 [file jmir_v14i3e84_app2.pdf]

Appendix 2: Adjacent areas to the sampled postcode areas

Table 2 shows adjacent areas to the sampled postcode areas.

A<br>**Adwords**	Adjacent areas	**B**<br>**Local websites**	Adjacent areas	C<br>**Adwords & local websites**	Adjacent areas	D<br>**Control**	Adjacent areas
**Liverpool**<br>(L)	Preston (PR)<br>Wigan (WN)<br>Warrington (WA)<br>Chester (CH)	**Leeds**<br>(LS)	Harrogate (HG)<br>Bradford (BD)<br>Huddersfield (HD)<br>Wakefield (WF)<br>York (YO)	**London SW**<br>(SW)	London (WC)<br>London(W)<br>London (SE)<br>London (EC)<br>Twickenham (TW)<br>Sutton (SM)	**Nottingham**<br>(NG)	Derby (DE)<br>Sheffield (S)<br>Doncaster (DN)<br>Lincoln (LN)<br>Peterborough (PE)<br>Leicester (LE)
**Redhill**<br>(RH)	Brighton (BN)<br>Guildford (GU)<br>Tonbridge (TN)<br>Kingston (KT)<br>Croydon (CR)<br>Sutton (SM)	**Southend**<br>(SS)	Romford (RM)<br>Dartford (DA)<br>Chelmsford (CM)<br>Medway (ME)<br>Bromley (BR)	**Kingston**<br>(KT)	Guildford (GU)<br>Twickenham (TW)<br>Sutton (SM)<br>Croydon (CR)<br>Redhill (RH)<br>Slough (SL)	**Oldham**<br>(OL)	Bolton (BL)<br>Manchester (M)<br>Stockport (SK)<br>Huddersfield (HD)<br>Halifax (HX)
**Lancaster**<br>(LA)	Carlisle (CA)<br>Darlington (DL)<br>Bradford (BD)<br>Blackburn (BB)<br>Preston (PR)<br>Blackpool (FY)	**Slough**<br>(SL)	Reading (RG)<br>Hemel Hempstead (HP)<br>Guildford (GU)<br>Twickenham (TW)<br>Southall (UB)	**Darlington**<br>(DL)	Carlisle (CA)<br>Newcastle (NE)<br>Durham (DH)<br>Cleveland (TS)<br>Harrogate (HG)<br>Lancaster (LA)<br>York (YO)	**Dudley**(DY)	Shrewsbury (SY)<br>Wolverhampton (WV)<br>Walsall (WS)<br>Birmingham (B)<br>Worcester (WR)
**Harrogate**<br>(HG)	Bradford (BD)<br>Darlington (DL)	**Kirkwall**<br>(KW)	(Aberdeen)<br>Inverness	**Shetland**<br>(ZE)	Kirkwall(KW)	**Hebrides**<br>(HS)	Inverness (IV)

	Leeds (LS)<br>York (YO)		Lerwick (ZE)				
