# Supplementary material for: Accuracy of Geographically Targeted Internet Advertisements on Google Adwords for Recruitment in a Randomized Trial
Source: J Med Internet Res. 2012 Jun 20;14(3):e84. doi: 10.2196/jmir.1991 (PMC3414907; doi:10.2196/jmir.1991)
Supplement: Supplementary file 3 [file jmir_v14i3e84_app3.pdf]

Appendix 3: Further information on boundaries

Figures 2-5 show hand-drawn polygons for four areas.

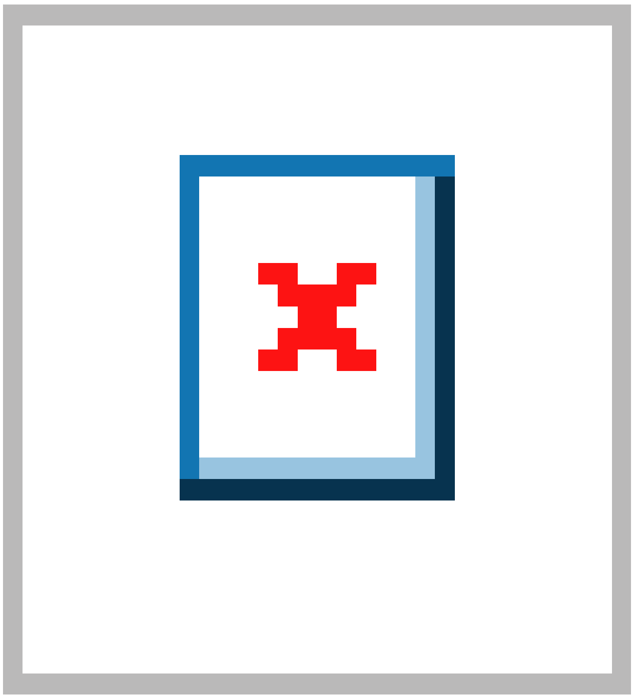

Figure 2. Polygon for Liverpool (L)

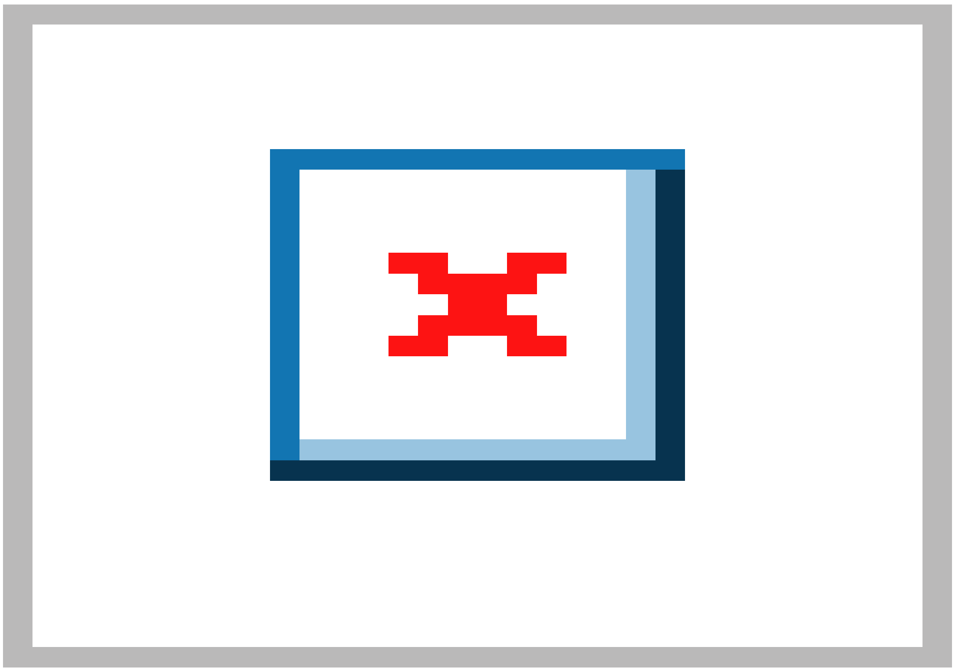

Figure 3. Polygon for Lancaster (LA)

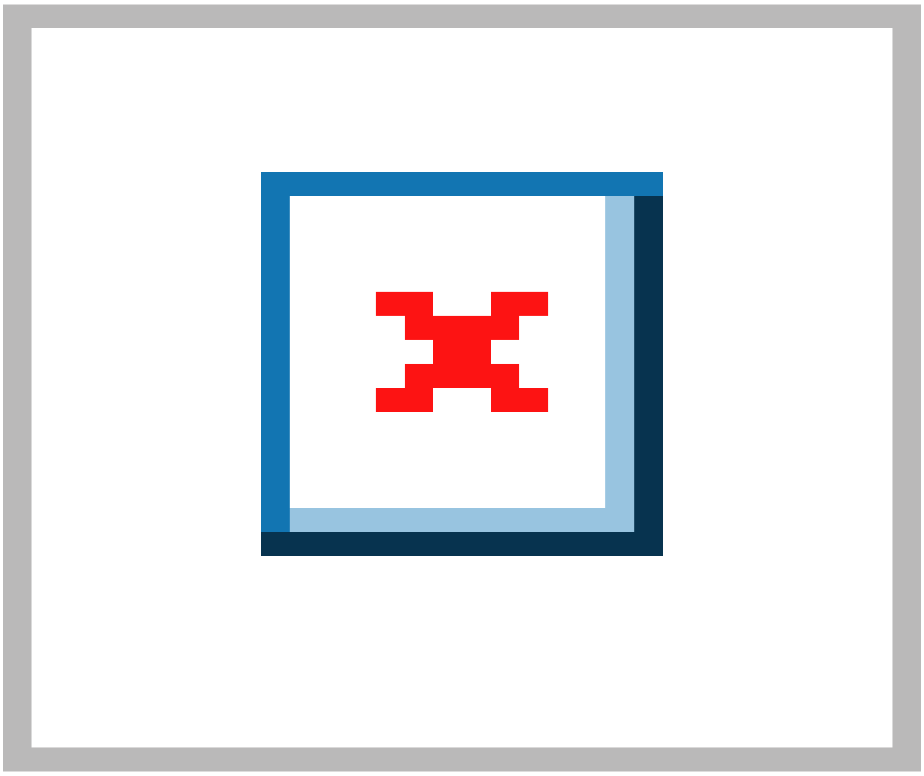

Figure 4 Polygon for Darlington (DL)

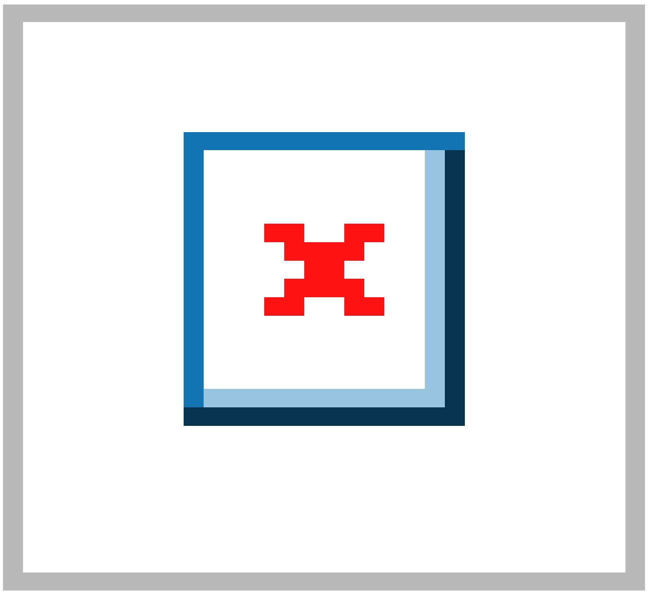

Figure 5. Polygon for Shetland (ZE)

<u>The leakage problem: further exploration of London SW</u>

Google is not particularly helpful in giving details of how it targets its Adwords. We targeted by using its one mile radius around a postcode district (e.g. SW1). There was no facility to target a postcode area (e.g. SW). So for each of our postcode areas we targeted one mile circles around each of the component postcode districts. To explore whether this method was likely to explain the leakage to neighbouring postcode areas we examined the SW postcode area in more detail.

Google maps places its marker (Figure 6) for SW1 at SW1E 5ER (found by zooming down on Google Maps, finding address from street level view and then using the post office postcode finder http://www2.royalmail.com/postcode-finder). We do not know if Adwords centres on the same location but in the absence of better information we have used this method.

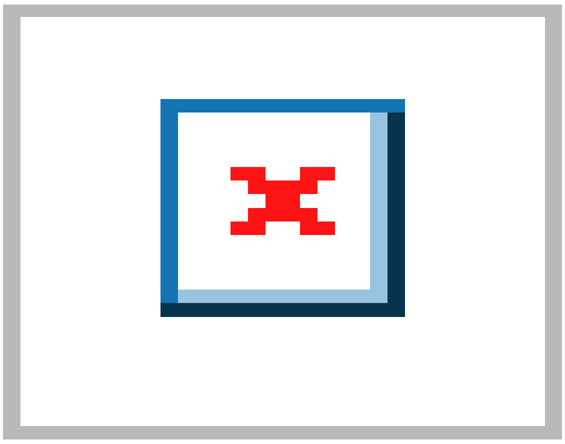

Figure 6. The location marker for London SW1 from Google Maps.

If we then use the radius plotter at http://www.freemaptools.com/radius-from-uk-postcode.htm we can see one mile around SW1 (Figure 7).

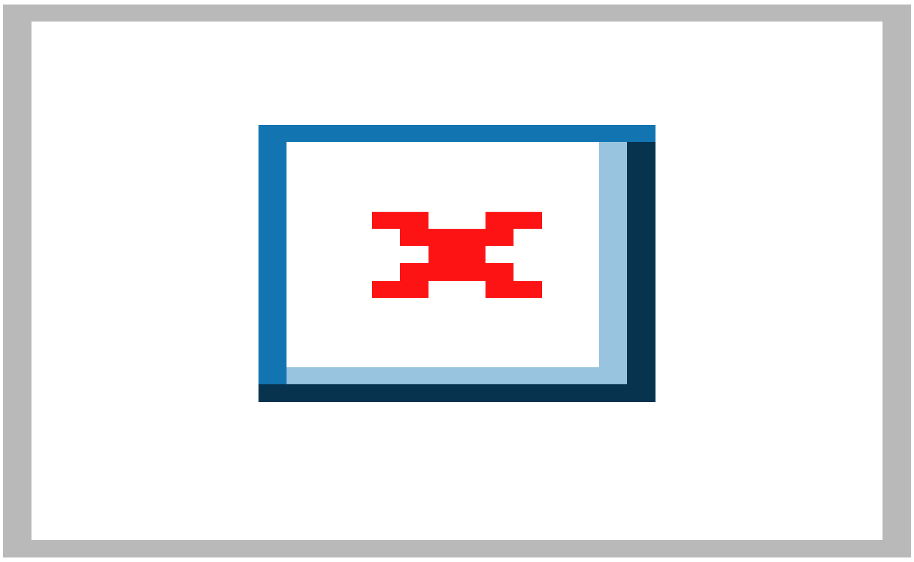

Figure 7. 1 mile radius around SW1.

Taking this approach we identified 20 addresses from Google Maps for each of the 20 postcode districts, found their postcodes from postcode finder (Table 3) and plotted the one mile circles (Figure 8).

SW1	1 Warwick Row, SW1E 5ER	SW7	1 Cromwell Mews, SW7 2JS	SW13	9 Lowther Road SW13 9NX

SW2	Warnham House, Upper Tulse Hill, SW2 2SA	SW8	57 Thorparch Road SW8 4RH	SW14	3 Richmond Park Road SW14 8JU
SW3	51, Elystan Street, SW3 3NY	SW9	St George's Primary School, Corunna Road, SW8 4JS	SW15	4 Heathview Gardens SW15 3SZ
SW4	The Pavement SW4 0HY	SW10	29 Fernshaw Road, SW10 0TG	SW16	496 Streatham High Road, SW16 3QB
SW5	30 Earls Court Square, SW5 9DQ	SW11	10 Falcon Lane, SW11 2LG	SW17	139 Fishponds Road SW17 7LL
SW6	602 Fulham Road, SW6, 5PA	SW12	55 Ormley Road, SW12 9QF	SW18	65 Twilley Street, SW18 4NU
				SW19	23 St Aubyn's Avenue, SW19 7BL
				SW20	60 Grand Drive, SW20 9DY

Table 3. Addresses and postcodes of Google map markers for the 20 SW postcodes.

The twenty one mile circles are shown in Figure 8 and appear to correspond quite well with the overall postcode area of SW (Figure 9) taken from

www.freemaptools.com/uk-postcode-map.htm

.

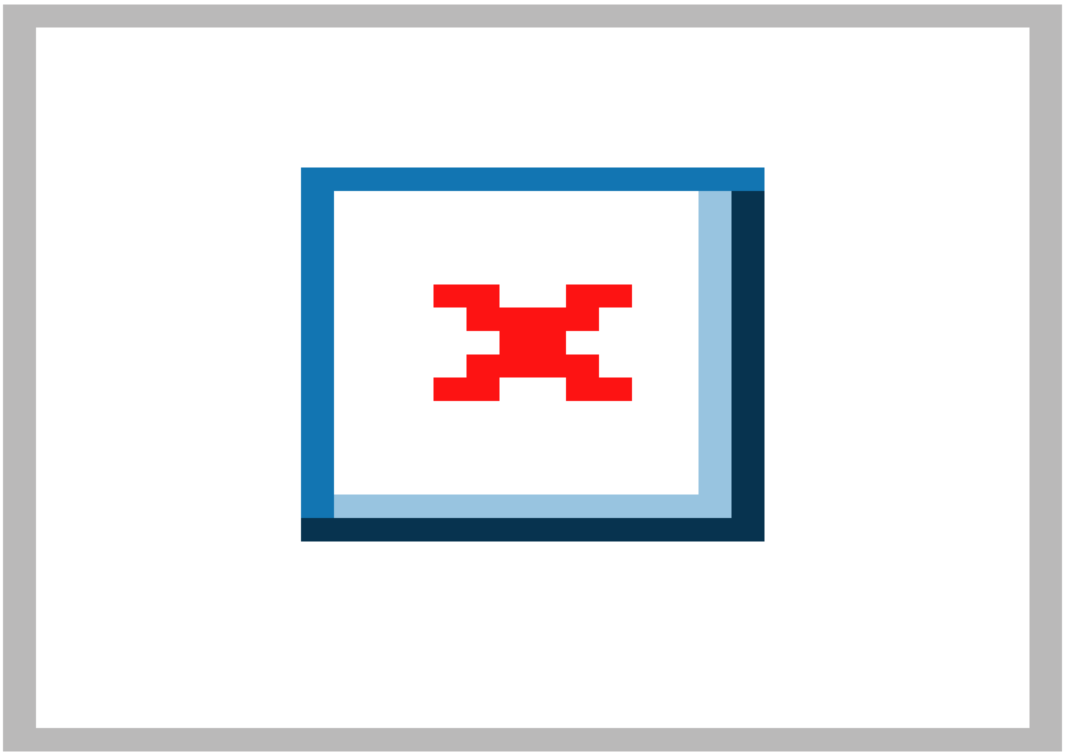

Figure 8.One mile circles for the 20 SW postcode districts.

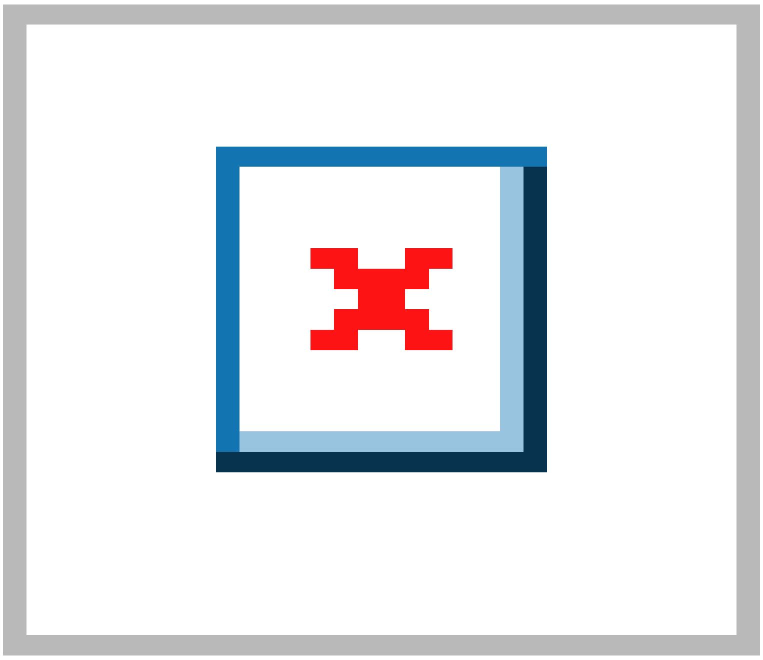

Figure 9. Boundary of SW postcode area.

Looking at the north-west corner of the area in more detail (below) (Figures 10 and 11), we can see that the ‘leakage’ (in theory) from this method to neighbouring postcode areas is very small.

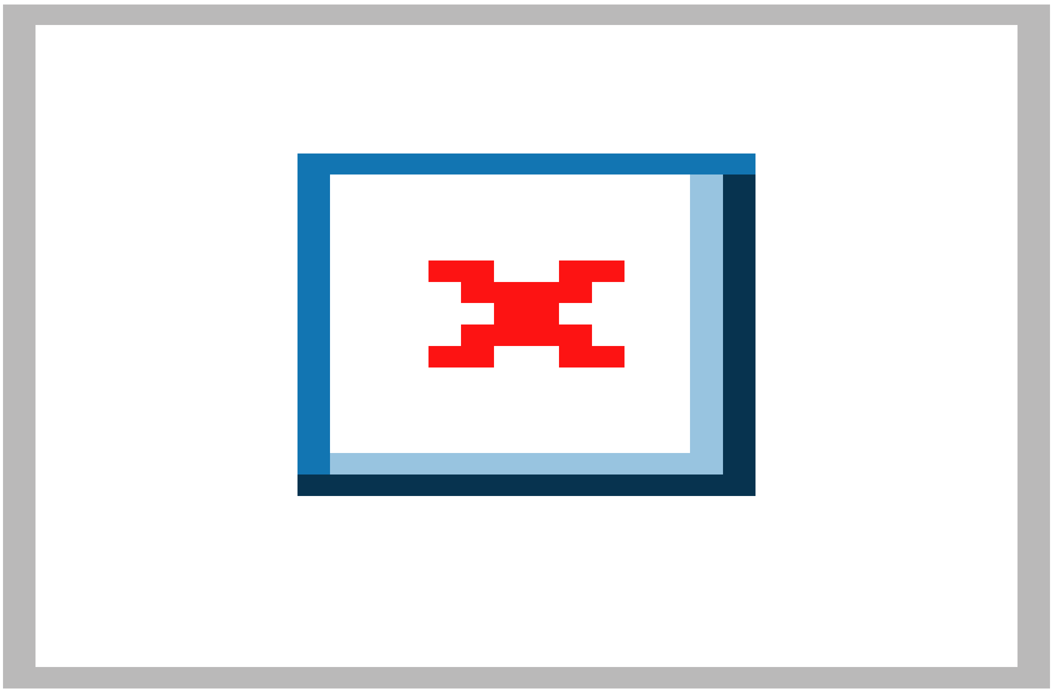

Figure 10. One mile circles in north-west corner of London SW.

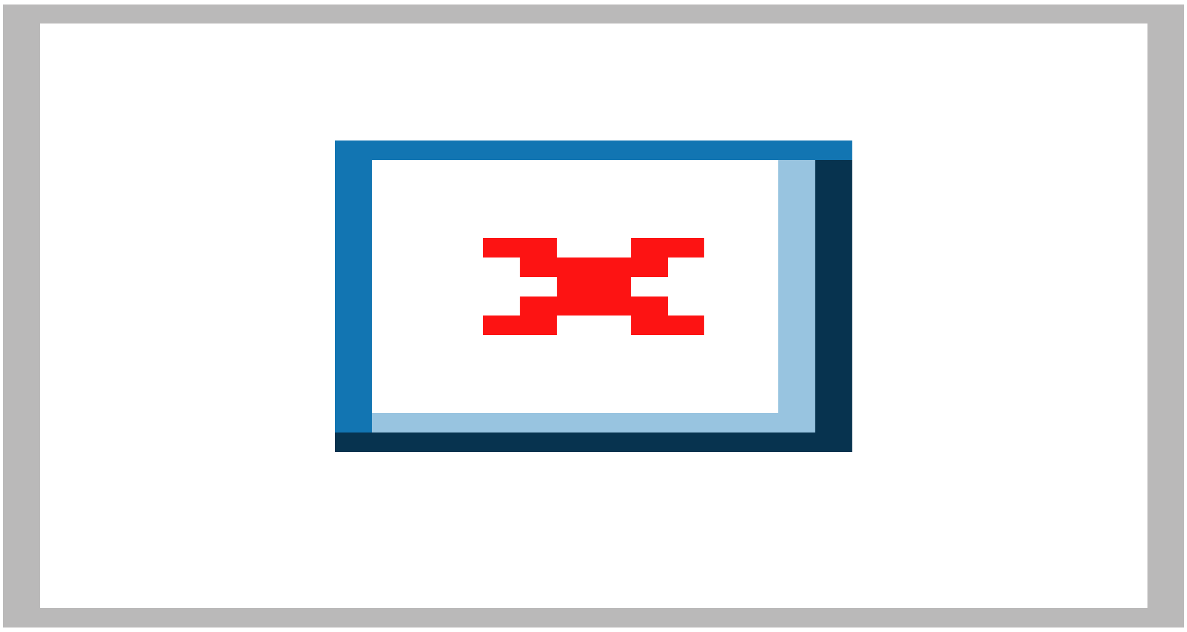

Figure 11. Postcode area boundary of the north-west corner of London SW.
