# Supplementary material for: Accuracy of Geographically Targeted Internet Advertisements on Google Adwords for Recruitment in a Randomized Trial
Source: J Med Internet Res. 2012 Jun 20;14(3):e84. doi: 10.2196/jmir.1991 (PMC3414907; doi:10.2196/jmir.1991)
Supplement: Supplementary file 4 [file jmir_v14i3e84_app4.pdf]

**Appendix 6: Keywords**

The following are the keywords used by Google Adwords. We started by entering ‘depression’ and Adwords suggested other keywords. We ‘accepted all’.

depression
suicide
depression help
samaritans
dealing with depression
symptoms depression
treatment for depression
pissed off
clinical depression
depression and anxiety
clinical depression symptoms
treatment depression
depression anxiety
how to deal with depression
cure for depression
symptoms for depression
depression anxiety symptoms
treatments for depression
depression treatment
self help depression
depression support
how to help with depression
anxiety depression
help with depression
clinic depression
depression with anxiety
depression self help
herbal remedies for depression
help for depression
vitamins for depression
help depressed
ect treatment for depression
natural remedies for depression
support groups for depression
depression and suicide
self help for depression
curing depression
deal with depression
how to help depression

remedies for depression
therapy for depression
helping depression
cures for depression
depression self help groups
depression treatments
herbs for depression
depression help groups
ways to deal with depression
treatment for depression and anxiety
foods to help depression
depression support groups
natural cures for depression
best treatment for depression
depression cures
depression and anxiety help
foods that help depression
depression remedies
helping with depression
shock treatment for depression
depression and anxiety treatment
support groups depression
depression self help guide
natural remedy depression
alternative treatments for depression
natural treatment for depression
holistic treatment for depression
help groups for depression
herbal remedies depression
online help for depression
depression support group
depression natural remedies
dealing with depression and anxiety
clinical depression treatment
christian help for depression
homeopathic remedy for depression
help for depression and anxiety
natural depression remedies
help with depression and anxiety
herbal treatment depression
symptoms of depression and anxiety
depression and anxiety symptoms
need help with depression
homeopathic remedies for depression
