# Supplementary material for: Accuracy of Geographically Targeted Internet Advertisements on Google Adwords for Recruitment in a Randomized Trial
Source: J Med Internet Res. 2012 Jun 20;14(3):e84. doi: 10.2196/jmir.1991 (PMC3414907; doi:10.2196/jmir.1991)
Supplement: Supplementary file 5 [file jmir_v14i3e84_app5.pdf]

Appendix 4: Screen shots from research website

Our original design was just to offer the two online sites but the ethics committee asked for Samaritans to be included so to have a balanced display (4 links) we also offered an information site NHS Choices.

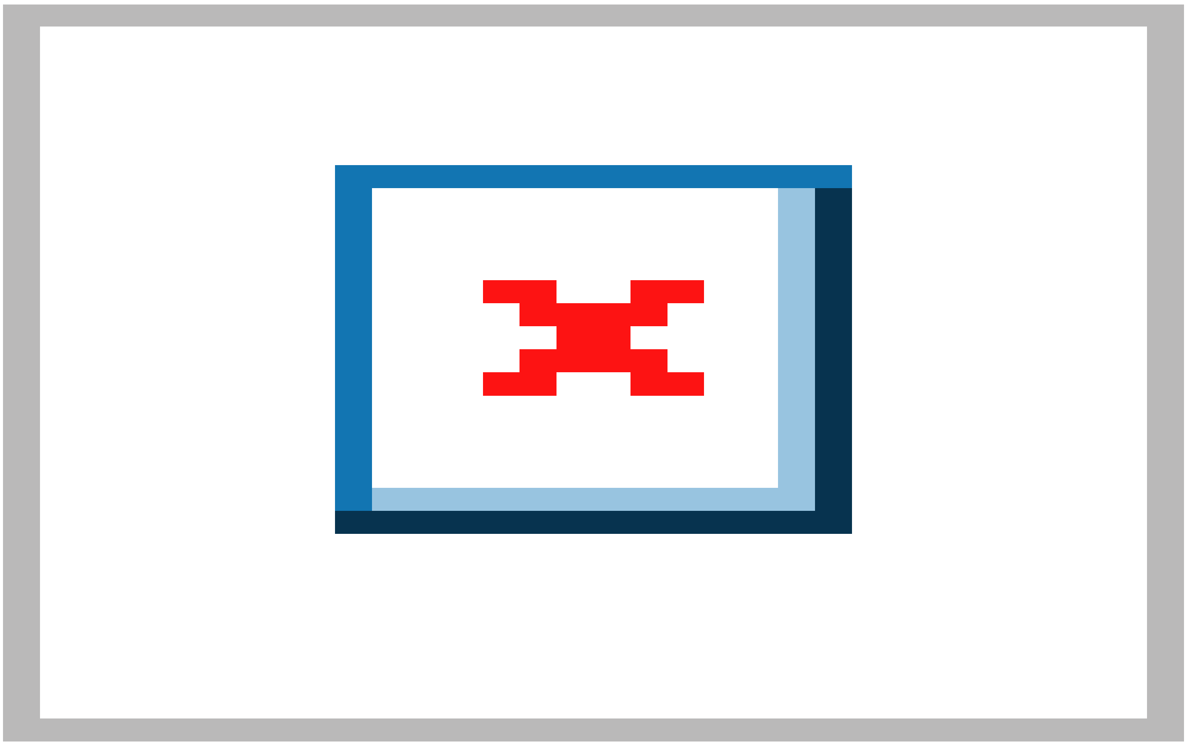

Figure 12. First screenshot from website

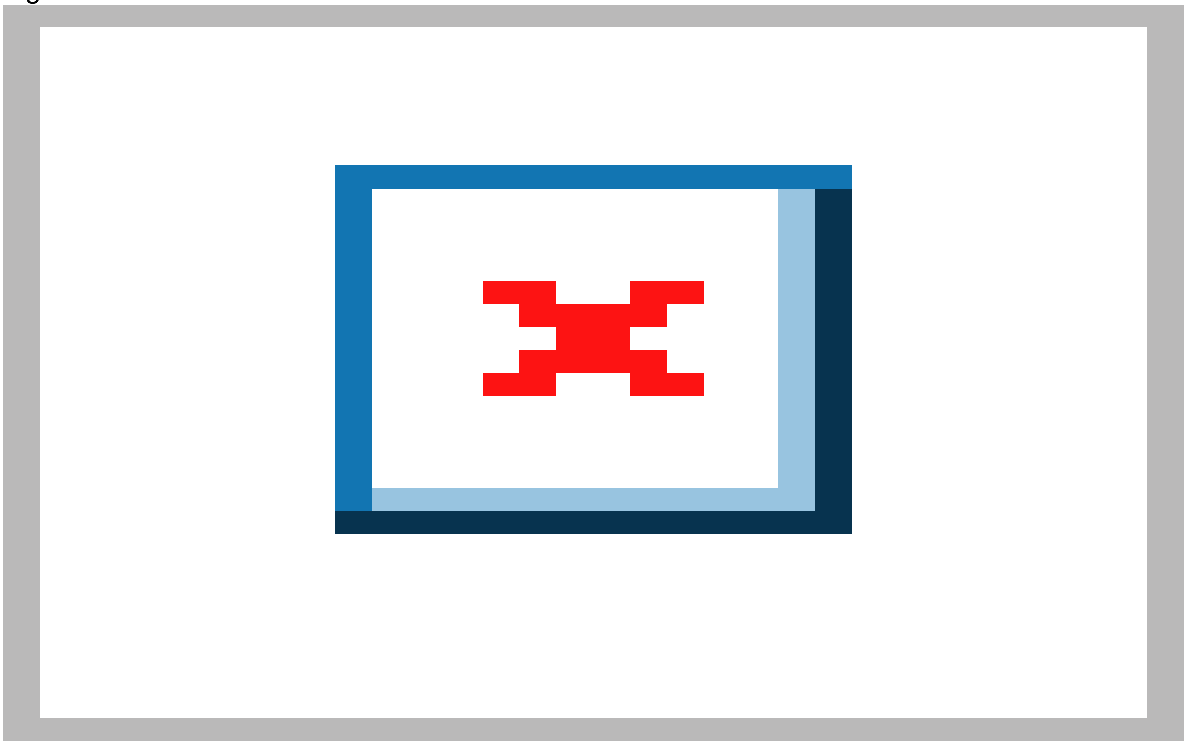

Figure 13. Second screenshot from website

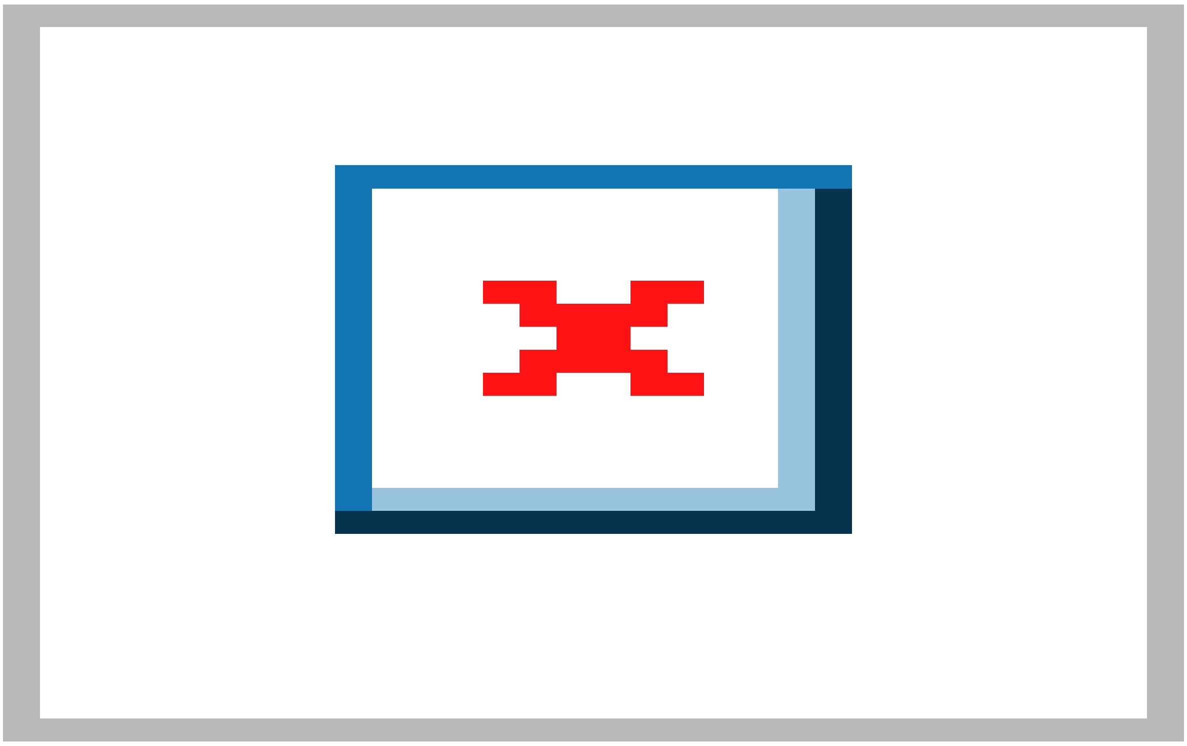

Figure 14. Third screenshot from website

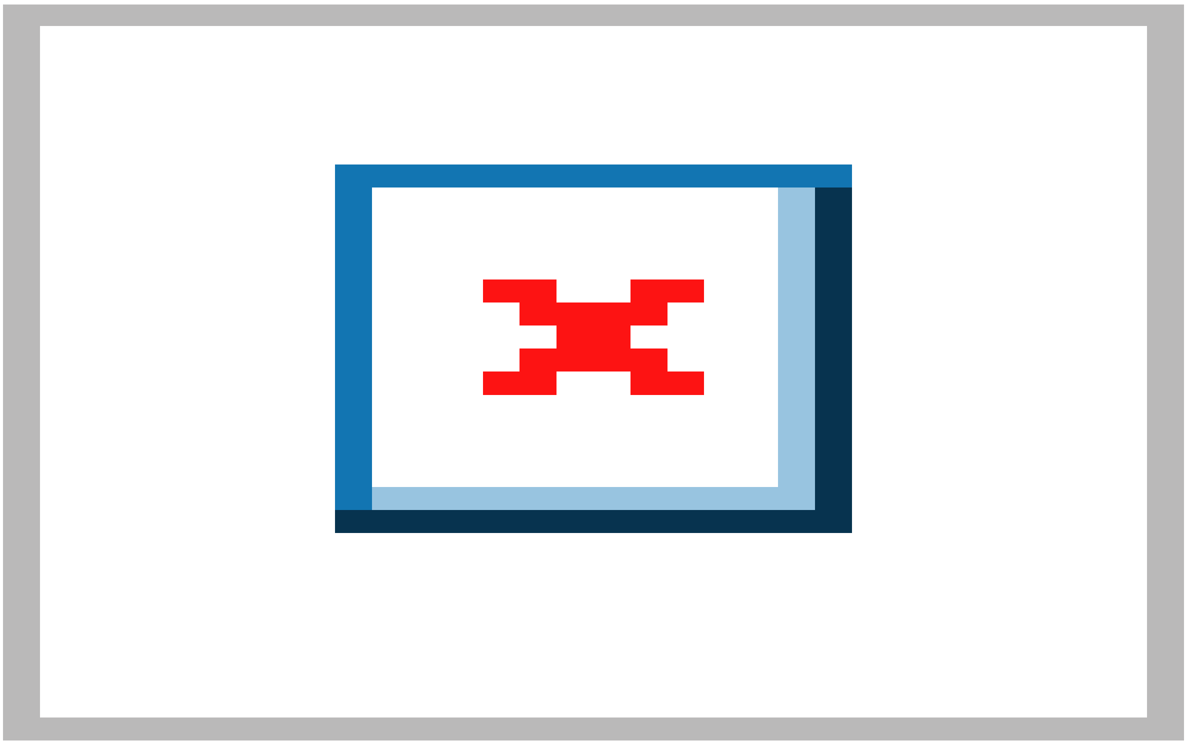

Figure 15. Fourth screenshot from website

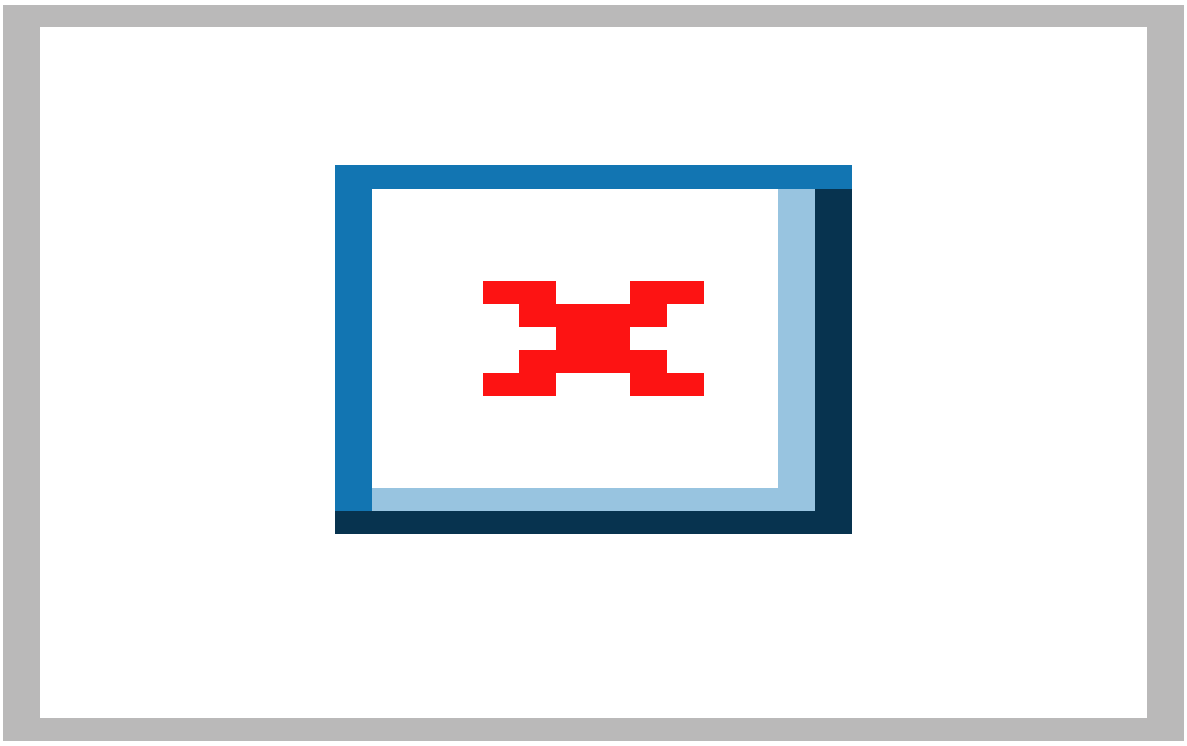

Figure 16. Fifth screenshot from website
