# Supplementary material for: Accuracy of Geographically Targeted Internet Advertisements on Google Adwords for Recruitment in a Randomized Trial
Source: J Med Internet Res. 2012 Jun 20;14(3):e84. doi: 10.2196/jmir.1991 (PMC3414907; doi:10.2196/jmir.1991)
Supplement: Supplementary file 6 [file jmir_v14i3e84_app6.pdf]

Appendix 5: Change made as a result of the pilot period

Initially we asked for postcode area without giving clarifying ‘towns’ but noted that quite a few people with Liverpool and Lancaster IP locations gave NW as their postcode, probably thinking of NW England rather than London NW. We added the postcode town to the question for the data analysed in this paper (see screen shots Appendix 4).
