# Supplementary material for: Accuracy of Geographically Targeted Internet Advertisements on Google Adwords for Recruitment in a Randomized Trial
Source: J Med Internet Res. 2012 Jun 20;14(3):e84. doi: 10.2196/jmir.1991 (PMC3414907; doi:10.2196/jmir.1991)
Supplement: Supplementary file 7 [file jmir_v14i3e84_app7.pdf]

Appendix 7

	Population	Budget	Clicks	Impressions	Click through rate
	**Main period 17th April to September 30th**				
All areas	3434112	£7.50	5350	392285	1.36
	**October 1st – November 30th**				
Liverpool	843450	£3.84	365	41649	0.88
London SW	783340	£3.56	886	76570	1.16
Redhill	494414	£2.24	182	8688	2.09
Kingston	490104	£2.22	174	17544	0.99
Darlington	341488	£1.56	66	4093	1.61
Lancaster	325972	£1.48	64	8075	0.79
Harrogate	133356	£0.60	21	1345	1.56
Shetland	21988	£0.60	1	62	1.61
All areas	3434112	£16.10	1759	158026	1.11

In the discussion we had put forward hypotheses to explain the imbalance between Arm C and Arm A. Arm C, which included London SW, had (according to Analytics) nearly four times the number of registrants on our website compared to arm A. The population of each was similar so either (i) AdWords showed the advert disproportionately (for a given number of people searching on ‘depression’) more often in London resulting in more hits, or (ii) there were more people in London searching on ‘depression’ resulting in AdWords displaying it more often, or (iii) more people in London clicked through on the advert than in other areas.

AdWords provides click through rates by campaign or by keyword but not by location within a campaign. As we had our AdWords campaign set up initially – as one campaign that included the various locations – we could not analyse click through rate by location. However, subsequent to the main analysis we were able to test this over two months. We set up separate AdWords. In retrospect, a better strategy to running one AdWords campaign campaigns for each of the eight postcode areas each with its own advert (as shown in the main paper Figure 6).

The Table above shows that we allocated a budget in proportion to the target population but increased the overall budget from £7.50/day to just over £16 per day. As can be seen, despite having a lower budget (£3.56 Vs £3.84) and lower population, London SW had nearly twice as many impressions as Liverpool. Click rate through rates were similar. This suggests that of our hypotheses either (i) and/or (ii) are true.
